# Tensile Deformation and Fracture of Unreinforced AZ91 and Reinforced AZ91-C at Temperatures up to 300 °C

**DOI:** 10.3390/ma16134785

**Published:** 2023-07-02

**Authors:** Nashmi H. Alrasheedi, Sabbah Ataya, Mohamed M. El-Sayed Seleman, Mohamed M. Z. Ahmed

**Affiliations:** 1 Department of Mechanical Engineering, College of Engineering, Imam Mohammad Ibn Saud Islamic University, Riyadh 11432, Saudi Arabia; nhalrasheedi@imamu.edu.sa; 2 Department of Metallurgical and Materials Engineering, Faculty of Petroleum and Mining Engineering, Suez University, Suez 43512, Egypt; mohamed.elnagar@suezuniv.edu.eg; 3 Mechanical Engineering Department, College of Engineering at Al Kharj, Prince Sattam bin Abdulaziz University, Al Kharj 11942, Saudi Arabia

**Keywords:** AZ91, composites, squeeze casting, carbon short fibers, tensile testing, flow curves, fracture

## Abstract

Magnesium alloys are still attractive materials for applications that necessitate light weight due to their low density, moderate strength, and good corrosion resistance. AZ91 is one of the widely applied magnesium alloys due to its very good castability and strength. However, one of the drawbacks of magnesium alloys is the low elastic modulus. So, reinforcing AZ91 with carbon short fibers with the aim of further increasing the strength and improving the elastic modulus is investigated in this study. Squeeze cast AZ91-23 vol.% carbon short carbon (AZ91-C) and the unreinforced AZ91 are deeply examined by tensile testing at different temperatures (20, 100, 150, 200, 250, and 300 °C). Tensile stress–strain curves are measured and the tensile parameters (yield stress, ultimate tensile strength and strain) are defined and presented against the test temperature. Yield stress of AZ91 at 20 °C (109 MPa) is doubled (226 MPa) in the reinforced AZ91-C. Yield stress is found to slightly decrease with increasing the test temperature. Ultimate tensile strength of AZ91 at 20 °C (198 MPa) is increased (262 MPa) in the reinforced AZ91-C. The improvement of the ultimate tensile strength due to reinforcing increases with increasing the test temperature. Flow curves are determined and described by a modified Mecking–Kocks relationship and the flow parameters are determined and described as a function of the test temperature. Microstructure investigation was undertaken of the fractured tensile specimens at the grain boundaries rich in eutectic structure formed at the grain boundaries. Mixed brittle/ductile fracture mode is detected on the fracture surface of unreinforced AZ91, while the SEM investigations show matrix/carbon fiber detachment and fiber fracture as main fracture modes.

## 1. Introduction

Among many types of magnesium alloys that have been developed, the AZ91 magnesium alloy is widely applied with the following approximate composition (in wt%): 9% aluminium (Al), 1% zinc (Zn), and 0.2% manganese (Mn) [1]. AZ91 has been widely used in automotive parts such as engine cradle, pedal, and gearbox housing [2]. The addition of Al and Zn to magnesium has been proven to improve its casting ability and strength, while Mn is added with the aim of increasing its ductility. However, this AZ91 magnesium alloy still exhibits relatively poor corrosion resistance, low stiffness, and strength at low and high temperatures [3]. Instead, several methods have been used to improve the properties and performance of AZ91 magnesium alloy for use in specific industrial fields. These include engineering manufacturing processes such as extrusion [4], severe plastic deformation [5], friction stirring [6], microstructure engineering using heat treatment techniques [7,8], and so on. It is not rare to combine two or more methods to obtain the expected criteria and strength that can be applied more widely. For example, Tan et al. [9] have examined the strength of AZ91 magnesium alloy processed by hot extrusion and followed by a series of different heat treatments. The results showed that the as-extruded AZ91 alloy followed by solution treatment experienced a significant increase in ductility, but at the same time, the tensile strength was not much different from the previous one (i.e., without solution treatment). On the other hand, the hardness of the as-extruded AZ91 alloy decreased drastically after solution treatment and subsequent aging treatment. Turning to the as-extruded AZ91 alloy subjected to annealing treatment, its tensile strength decreased and, concurrently, its ductility increased considerably.

Furthermore, diligent research has been carried out with an emphasis on enhancing the performance of magnesium alloys at high temperatures, particularly on the issue of their mechanical properties. Among the approaches that can be taken to achieve the above intention are (i) the addition of appropriate alloying elements [10,11,12]; (ii) the reduction of grains to ultrafine sizes [13], and (iii) the development of magnesium alloy-based composites [14]. For example, Zhang et al. [15] investigated the effect of adding minor Sr elements with Sr concentrations of 0, 0.2, 0.6, and 1.0 wt% on the mechanical properties and creep behavior of a high-pressure die-casting AZ91-0.5RE-based alloy. The results obtained show that the addition of 0.2% Sr succeeded in significantly improving the mechanical properties of the AZ91-0.5RE-based alloy at ambient temperature. Meanwhile, the fracture surfaces of samples with and without Sr addition exhibited ductile fracture and quasi-cleavage fracture types, respectively. In fact, Chen et al. [15] studied the tailoring microstructure evolution and fracture damage behavior of a Mg-Y-Zn alloy during hot tensile deformation with a temperature range between 250 and 400 °C and a strain rate of 0.005–0.1 s^−1^. The important result was that the hot tensile strength and flow stress decreased with increasing tensile temperature or decreasing strain rate. Meanwhile, the fracture strain demonstrated an abnormal trend under the influence of different hot tensile temperatures and strain rates, which is due to the dynamic softening and damage in the interior material caused by the work-hardening combination. Shastri et al. [16] investigated the relationship between microstructure and tensile and creep properties of the AZ91 alloy by three casting methods, that is, gravity casting (GC), squeeze casting (SC), and high-pressure die-casting (HPDC). The previous results indicate that the yield stress and ultimate tensile strength decreased, and as a consequence, ductility increased for all samples when examined at temperatures of 150 and 200 °C. Whereas, the ductility of GC, SC, and HDPC samples was increased by 21%, 14.7%, and 15.7% at 150 °C, and by 26.3%, 25%, and 20.6% at 200 °C, respectively. Additionally, Xiao et al. [17] evaluated the effect of Ca content and rheo-squeeze casting parameters on the microstructure and mechanical properties of the AZ91-1Ce-xCa alloy. The experiments conducted show that an increase in the rotational speed and applied pressure to 120 r/min and 130 MPa respectively resulted in an enhanced microstructure; furthermore, the ultimate tensile strength, elongation, and yield strength of the AZ91-1Ce-2Ca alloy reached optimum values of 189.1 MPa, 130.1 MPa, and 2.4%, respectively. However, although many studies have been undertaken to improve the strength and properties of the AZ91 alloy, the results so far are still unsatisfactory. Thus, it is crucial to keep conducting research that can assist in improving the performance of the AZ91 magnesium alloy, which has restricted its use in high-temperature components. 

Minor additions of some elements to Mg and Mg-alloys enhances new properties and applications of such alloys. For example, the addition of Gd to 0.5 Ca containing Mg alloy [18] improved the cytocompatibility of these alloys due to increasing the stability of these alloys which makes it competing for some biomaterials [19].

The approach taken in this study in order to improve mechanical properties is through the material transformation from magnesium alloys to magnesium composites. Several studies have noted that the addition of reinforcing materials has successfully improved mechanical strength, wear resistance, elastic modulus, and so on [20,21]. Among the materials that have been utilized as reinforcement for magnesium alloys are SiC [22], alumina [23], carbon nanotubes [24], tungsten disulphides [25], titanium carbide [26], and titanium diboride [27]. With regard to the fatigue behavior of magnesium composites, the addition of SiCp reinforcement by 20 or 25% by volume with a particle size of about 15 μm successfully improved the fatigue performance with respect to monolithic AZ91D [28]. In the meantime, Llorca et al. [29] mentioned that the addition of 0.25% volume of short alumina fibers to the AZ91 alloy was able to increase the fatigue strength by 85%. This was attributed to the fatigue crack initiation resistance of AZ91 composites being higher than that of AZ91 alloy alone. 

To date, from the literature search, it is still very rare to find research on the behavior of mechanical properties, especially tensile strength at high temperatures of AZ91 magnesium composites reinforced with short carbon fibers. Obviously, this is an attraction to research it further. In the present study, the AZ91 alloy was reinforced with short carbon as it is established from previous results that the addition of short carbon fibers is very effective in increasing the strength, wear resistance, and hardness of the AZ91 alloy [14,30]. Therefore, the main objectives of this work are: (1) to present lightweight and high- reinforcement volume fraction (23%) composite material with improved tensile properties, (2) to model the tensile deformation and flow behavior of the cast Mg-alloy AZ91 and present the description parameters for researchers for FE simulation tasks, and (3) to present the role of carbon short fibers and their orientation on the fracture behavior of the AZ91-C composites.

## 2. Materials and Methods

The test materials used in this study were unreinforced AZ91 and reinforced AZ91 (AZ91-C). The reinforcing materials used were short carbon fibers (L_f_ = 100 μm and d_f_ = 7 µm) with a high volume fraction of about *V*_f_ = 0.23 distributed quasi-isotropically in the AZ91 alloy matrix. Subsequently, the general properties of the AZ91 alloy acting as the matrix and the short carbon fiber acting as the reinforcing material are presented in Table 1 and the chemical composition of the AZ91 alloy is represented in Table 2.

**Figure 1:**
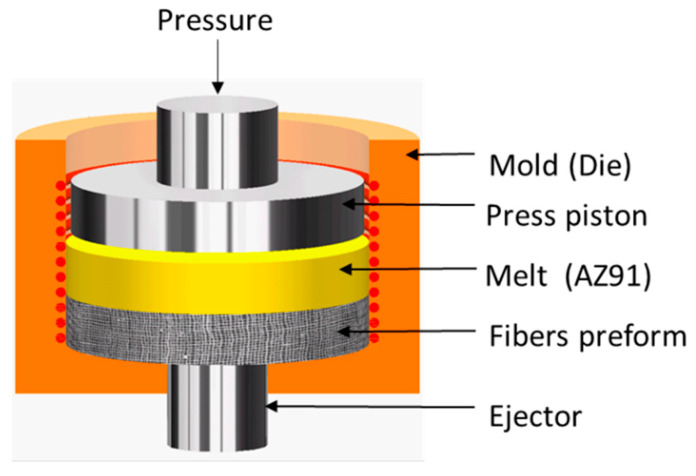
Schematic representation of the squeeze casting process used to produce fibers reinforced AZ91.

The composite material based on the AZ91 alloy reinforced with short carbon fibers was manufactured by squeeze casting, as shown in Figure 1. A preform was prepared from carbon fibers and placed in the mold and preheated to 400 °C. The molten material (AZ91) was poured at a superheating temperature of 730 °C to enhance the fluidity and penetration through the fibers preform. Subsequently, the melt is compacted by a piston using a squeezing pressure of 80 MPa. The squeeze cast composite was solidified at a rapid cooling rate, reaching around 28.2 °C s^−1^. More detailed information about this squeeze casting production technique can be found in the following descriptions [14,30,32].

**Figure 2:**
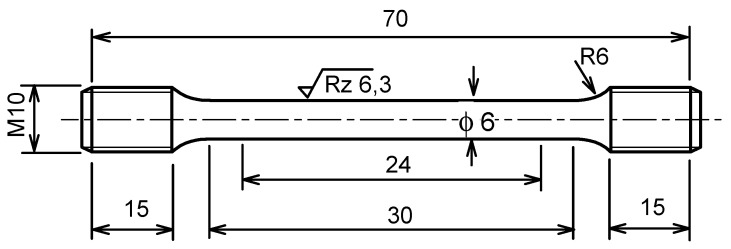
Tensile test specimen according to ASTM E8, adapted from ref. [33].

For the tensile test, the specimens used in this study were prepared by machining from the squeeze cast block. The detailed dimensions of the tensile test specimen refer to the ASTM E8 standard [33], as can be seen in Figure 2. A universal servohydraulic tensile testing machine of type MTS 810 (MTS Systems Corporation, Eden Prairie, MN, USA) with a maximum load capacity of 100 kN and a maximum speed of 100 mm/s was used to conduct the tensile tests. In addition, the machine is equipped with a furnace that can be heated to a maximum temperature of approximately 800 °C. The tensile test conditions applied in this experiment were a strain rate of 0.001 s^−1^ and a crosshead speed of 0.03 mm/s. Furthermore, the tensile tests were carried out at varying temperatures of 20, 150, 200, 250, and 300 °C. With regard to the length increase in the sample, it was measured using an extensometer with an accuracy of 0.5 µm with a maximum distance of 10 mm for testing at room temperature, while an extensometer with an inductive rod and an accuracy of 1 µm and a maximum distance of 40 mm was used for testing at high temperatures.

The microstructure of the as-cast materials and the longitudinal sections of the fractured tensile specimens have been investigated. Selected samples for optical microscopy were molded in epoxy forms for easy handling then subjected to grinding on SiC papers from grit size of 400 to 2000, then polished on cloth with the help of 0.04 µm alumina particles. Polished samples were etched using 2% Nital (2 mL HNO_3_, 23 mL water, 75 alcohol) for 15 s. Samples were good water washed followed by ethyl alcohol and dried after each stage. The microstructure was investigated by the digital optical microscope Type Leica DM4000M (Leica Microsystems GmbH, Wetzlar, Germany). Moreover, the fracture surfaces of the tested tensile samples were analyzed using scanning electron microscopy (SEM) with an LEO 1450 VP Type (Carl Zeiss, Jena, Germany) equipped with energy dispersive spectroscopy (EDS), Oxford Type (Oxford Instruments, Oxford, U.K.) with a voltage of 30 kV. SEM images were recorded using a secondary electron beam which enables the capturing of images of fracture surfaces at very good contrast, and fracture surfaces were gold spattered for improved image quality.

## 3. Results and Discussion

### 3.1. Initial Microstructure

**Figure 3:**
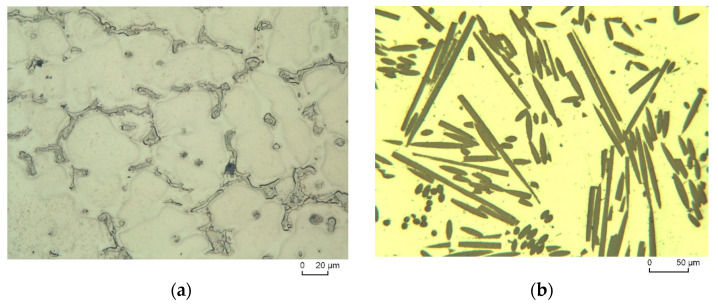
Optical microstructure of AZ91 and distribution of the fibers in AZ91-C. (**a**) The unreinforced alloy AZ91; (**b**) Distribution of fibers in AZ91-C.

Figure 3a shows the casting structure of AZ91. A massive eutectic structure from the β−phase Mg_17_(Al)_12_ and MgZn [16] is mainly found at grain boundary triple points. The remainder of the Al additive forms an α–Mg mixed crystal with magnesium and this is fit to [16]. The AZ91 alloy is a type of magnesium alloy having high strength, excellent castability, and is notably cheaper. On the other hand, this alloy has low creep resistance, especially at temperatures above 140 °C. Despite the low creep resistance, AZ91 [34] was chosen as one of the piston composite matrix alloys because it can be assumed that the reinforcement compensates for the low creep resistance. In terms of AZ91-C composites (Figure 3b), short carbon fiber-reinforcing materials are added quasi-isotopically with a uniform distribution in the reinforced plane of the AZ91 alloy. The distribution of short carbon fibers can be observed by cutting lengthwise or cross-sectioning the fibers. It is clearly seen that the short carbon fibers show relatively even distribution, good compatibility with the matrix, and good wettability with the melting process during the squeeze casting, which leads to the production of appropriate AZ91-C composites, as shown in Figure 3b. Low magnification was used in the image of the reinforced Mg alloy (Figure 3b) to show the distribution of the fibers, while higher magnification was applied for the image of the unreinforced AZ91 (Figure 3a) to show the cast microstructural details.

### 3.2. Tensile Testing Results

**Figure 4:**
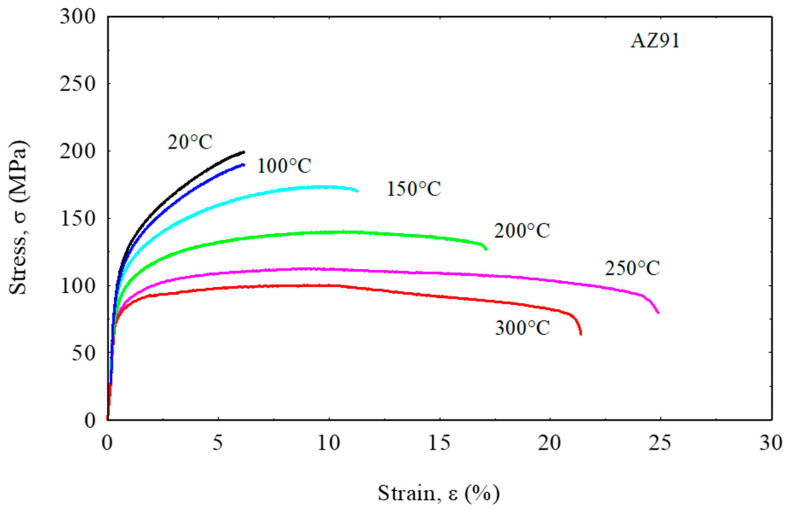
Tensile stress–strain curves of the unreinforced AZ91 at temperatures up to 300 °C.

Figure 4 shows the tensile stress curves of the unreinforced AZ91 at varying temperatures. In general, the tensile test results of the AZ91 alloy show a periodic strength decreasing trend with increasing tensile testing temperature. From the graph in Figure 4, it can be seen that the maximum tensile strength of about 198 MPa was achieved by the unreinforced AZ91 alloy at a tensile temperature of 20 °C, while the lowest tensile strength of about 90 MPa was achieved at a tensile temperature of 300 °C. More interestingly, the AZ91 alloy at temperatures of 20 and 100 °C shows a very rapid increase in flow stress at the initial stage and, concurrently, also shows brittle characteristics due to its relatively short elongation. In the case of an increase in the obtained tensile strength, it can be attributed to the work-hardening mechanism, which is the accumulation of dislocations and their degree of kinking [35] and it predominantly occurs at low temperatures [36]. As the test temperature increases, the AZ91 alloy becomes more ductile, as evidenced by the increasing elongation of the unreinforced AZ91 alloy, and conversely, its tensile strength decreases. The phenomenon of decreasing tensile strength in the unreinforced AZ91 alloy can be related to its softening behavior [37]. The softening phenomenon could be caused by microstructural softening that is triggered by deformation heating and by dynamic recrystallization [38].

**Figure 5:**
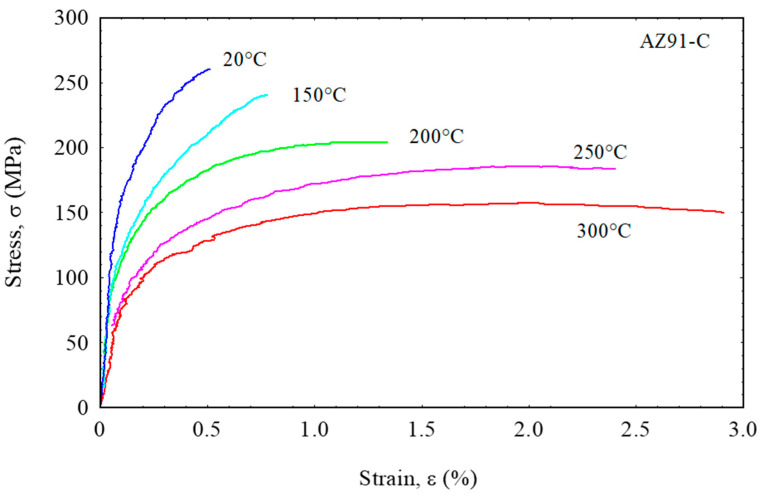
Tensile stress–strain curves of the reinforced AZ91 at temperatures up to 300 °C.

Figure 5 shows the relationship curves between stress and strain in the reinforced AZ91 at temperatures 20 °C to 300 °C. Encouragingly, the tensile strength of AZ91 reinforced with short carbon fibers showed a significant increase at all tensile testing temperatures, where the tensile strength of AZ91 composites was 265 MPa at 20 °C and 142 MPa at 300 °C. In terms of tensile testing temperature, the tensile strength of the reinforced AZ91 decreased gradually with increasing tensile testing temperature and showed a similar trend with the unreinforced AZ91. However, the tensile strength of the reinforced AZ91 is still superior to that of the unreinforced AZ91 at all testing temperatures. From these results, it can be understood that the addition of short carbon fibers has an important contribution to make in improving the mechanical properties of AZ91, where the short carbon fibers are able to resist the load transfer applied during tensile testing through interfacial shear stress and thus exert a strengthening effect on AZ91 composites [39]. Obviously, this strengthening effect is greatly influenced by the distribution properties and the formation of good bonding strength between short carbon fibers as reinforcing material and AZ91 as the matrix [40,41]. In previous work, it was reported that carbon fibers can help the solidification of a magnesium alloy matrix by acting as heterogeneous nucleation sites. The mechanical properties of the composite can also be improved by distributing carbon fibers at the grain boundary of the AZ91 alloy, where it is very effective in inhibiting grain growth [42,43].

**Figure 6:**
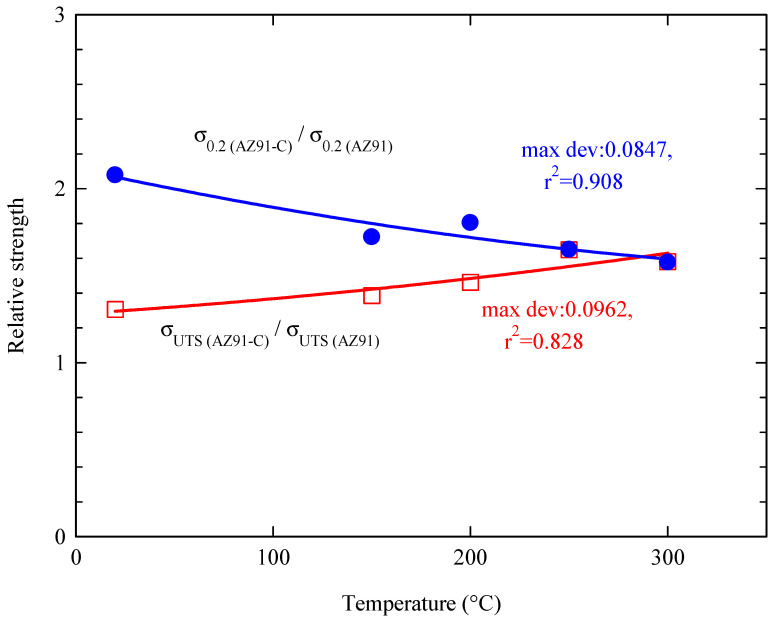
Relative tensile yield stress ($\sigma_{0.2\left(\mathrm{AZ}91-C\right)}\;/\sigma_{0.2\left(\mathrm{AZ}91\right)}$) and relative ultimate tensile strength ($\sigma_{\mathrm{UTS}\left(\mathrm{AZ}91-C\right)}/\sigma_{\mathrm{UTS}\left(\mathrm{AZ}91\right)}$ ) of the unreinforced and reinforced AZ91 at temperatures up to 300 °C.

Figure 6 shows the relative tensile yield stress and relative ultimate tensile strength, each obtained from the ratio between reinforced AZ91 and unreinforced AZ91 at varying temperatures, respectively. The relative tensile yield stress at 20 °C is the highest because the ratio between the yield stresses of the AZ91 composite and AZ91 alloy is more than two times (as shown in Figure 4 and Figure 5). Additionally, as the test temperature increases, the tensile yield stress of both reinforced AZ91 and unreinforced AZ91 decreases, indicating that the ratio also decreases simultaneously. On the other hand, the relative ultimate tensile strength value at 20 °C is the lowest because the ratio is only about 1.25. However, the ratio of ultimate tensile strength increases linearly with increasing the testing temperature because the reinforced AZ91 has better resistance to high temperatures compared to the unreinforced AZ91. This clearly indicates that short carbon fibers can improve the ultimate tensile strength of the AZ91 alloy [44]. Another thing that can be realized is that the relative tensile yield stress and the relative ultimate tensile strength have an inverse relationship. The obvious decrease in yield stress with increasing the test temperature for both unreinforced AZ91 and reinforced AZ91-C can be related to the affected metallic component (AZ91) by the increased test temperature. This effect represents a negative contribution of the matrix alloy in the reinforced AZ91 at a low range of strain which is around the yield stress range. At a higher degree of deformation beyond the yield stress, the fibers rotate and align with the loading direction showing higher deformation resistance and increased load-carrying capacity regardless of the test temperature. From a statistical perspective, the relative tensile yield strength has a slightly higher R^2^ value than the relative ultimate tensile strength, where the R^2^ value is a measure in statistics commonly used to describe how close the data are to the appropriate regression line.

Short carbon fibers in the preform used in squeeze casting of the reinforced AZ91-C are randomly oriented in a horizontal plane of the cylindrical cast part. The examined cylindrical test specimens were machined so the specimen axis was mostly in a radial direction of the cylindrical cast part. This arrangement makes the loading of the test specimens parallel to the reinforced plane to have a strengthening effect. Previous studies on AZ91-C [14,30] indicated that the achieved compressive strength [30] and wear resistance [14] were lower for samples loaded normal to the reinforced plane (parallel to the cylindrical cast part axis) than that for specimens loaded parallel to the reinforced plane.

**Figure 7:**
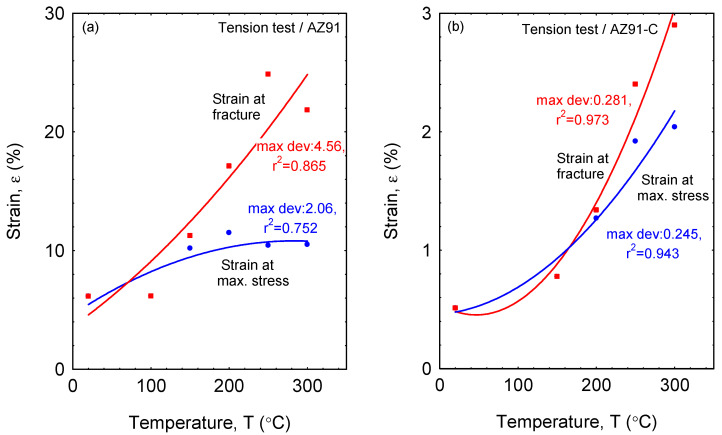
Tensile strain at fracture and strain at maximum stress at temperatures up to 300 °C for (**a**) unreinforced AZ91 and (**b**) reinforced AZ91-C.

Figure 7 illustrates the tensile strain at fracture and strain at maximum stress of unreinforced and reinforced AZ91 at temperatures up to 300 °C. In unreinforced AZ91, as can be seen in Figure 7a, the tensile strain at fracture increased significantly, proving that the increase in temperature greatly affects the increase in tensile strain at fracture, where at a temperature of 300 °C, the fracture tensile stress reaches about 25%. Turning to the strain at maximum stress, it only increased slightly from 20 °C to 200 °C, but after 200 °C, the strain at maximum stress plateaued. If looked at more carefully, the strain at maximum stress from temperatures of 150 °C to 300 °C is around 10% (Figure 7a). With regard to the reinforced AZ91, both the tensile strain at fracture and the strain at maximum stress show an increasing trend similar to the nonlinear function as a function of tensile testing temperature. Additionally, the difference between the two curves in Figure 7b lies in the strains achieved, where it appears that the tensile strain at fracture is larger than the strain at maximum stress with an increasing tensile testing temperature. This again refers to the important influence of the addition of short carbon fibers into the matrix, which has successfully increased the tensile strength of the AZ91 alloy. Hence, if observed closely in Figure 7, there is a sharp contrast in the difference in strain achieved by the two types of materials. The increased strain of unreinforced AZ91 with increasing the test temperature can be understood as most metallic alloys showing higher ductility and increased span between the strain at maximum stress and strain at fracture at a higher temperature. At this span, there is compensation between strengthening by deformation and softening by recrystallization and nucleation of voids as an initiation of damage features. This usual behavior of metallic materials has abridged in the reinforced AZ91 due to the reduced ductility affected by the addition of brittle long bodies which showed a low response to strain. Thus, it can be asserted here that composites have much better strength than their alloys [44,45].

**Figure 8:**
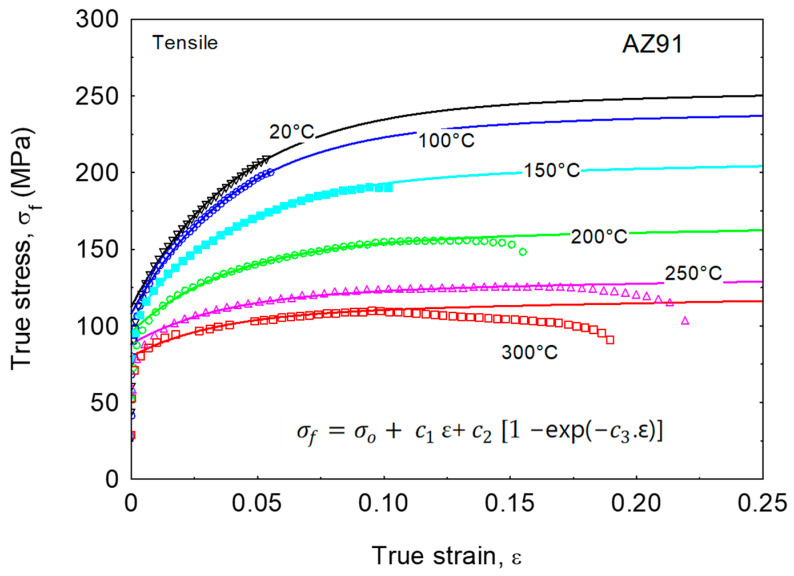
Description of the tensile true stress–true strain curves of the matrix alloy AZ91 at temperatures up to 300 °C.

Figure 8 shows the illustration of the tensile true stress–strain curves of an AZ91 matrix alloy at temperatures from 20 °C to 300 °C. When the AZ91 alloy was deformed at temperatures above 150 °C, strain softening occurred after the initial strain-hardening stage. While strain hardening is somewhat noticeable at all temperatures, the AZ91 alloy is deformed at a constant strain rate of 0.001 s^−1^ and is very obvious at a temperature of 20 °C. It is understood that the lower the temperature and the greater the applied strain level, the higher the flow stress level. The contention for this phenomenon is due to the formation of tangled dislocation structures which might serve as a barrier to dislocation movement, as higher strain levels are believed to be the main reason for the apparent results [46].

Modeling tensile stress–strain flow curves are of great importance by means of finite element simulation of components with the same material behavior, therefore, a description of the flow curves using a suitable material law and the determination of the materials’ parameters are sought in order to successfully model the flow behavior. In one study [47], there is a comparison of four empirical relationships for the description of flow curves of austenitic stainless steel at different temperatures in a relatively small range of deformation (up to 0.2). The Swift [48] and Voce [49] relationships were found to represent the low-strain flow curves well. A good description is also possible with the Ludwik relation if the parameter is chosen as any constant whose value is smaller than the yield point.

The flow curves from tensile tests on the magnesium alloys AE42 and AZ91 are described very well with the modified Mecking–Kocks relationship [47], (Equation (1)):

(1) $$\sigma_{f}=\sigma_{o}+C_{1}\varepsilon +C_{2}\left[1-exp\left(-C_{3}\epsilon \right)\right]$$

Other descriptions, for example, according to Swift, provide less accurate fitting results. This formula has been successfully used by Ataya and El-Magd [50] to describe the compressive stress-strain curves of the same material (AZ91) at temperatures up to 300 °C.

**Figure 9:**
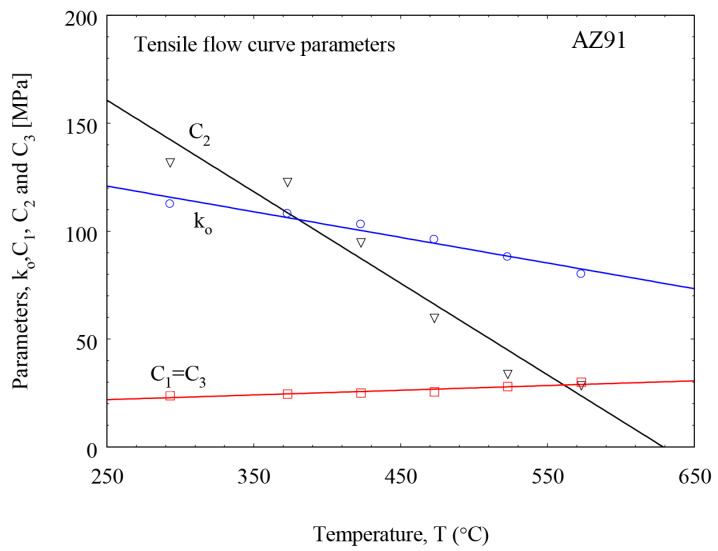
Description parameters of the tensile flow curves of the matrix alloy AZ91 at temperatures up to 300 °C.

It was found that the value of parameter $C_{3}$ can be set equal to $C_{1}$. This helps in lowering the number of fitting parameters. The material flow parameters (Table 3) $\sigma_{o}$, $C_{1}$ and $C_{2}$ used in Equation (1) are plotted in Figure 9 as a function of the tensile test temperature (in K) for AZ91. These parameters are linearized with Equation (2):

(2) $$\sigma_{o\;}\left(T\right)=k_{f}\left(T_{o}\right)\left(1-c\frac{\left(T-T_{o}\right)}{T_{m}}\right)$$

The constants of Equation (2) are listed in Table 2, where *T_o_* = 273 K and *T_m_* = 873 K.

### 3.3. Optical Microscopy of Fracture Surface

**Figure 10:**
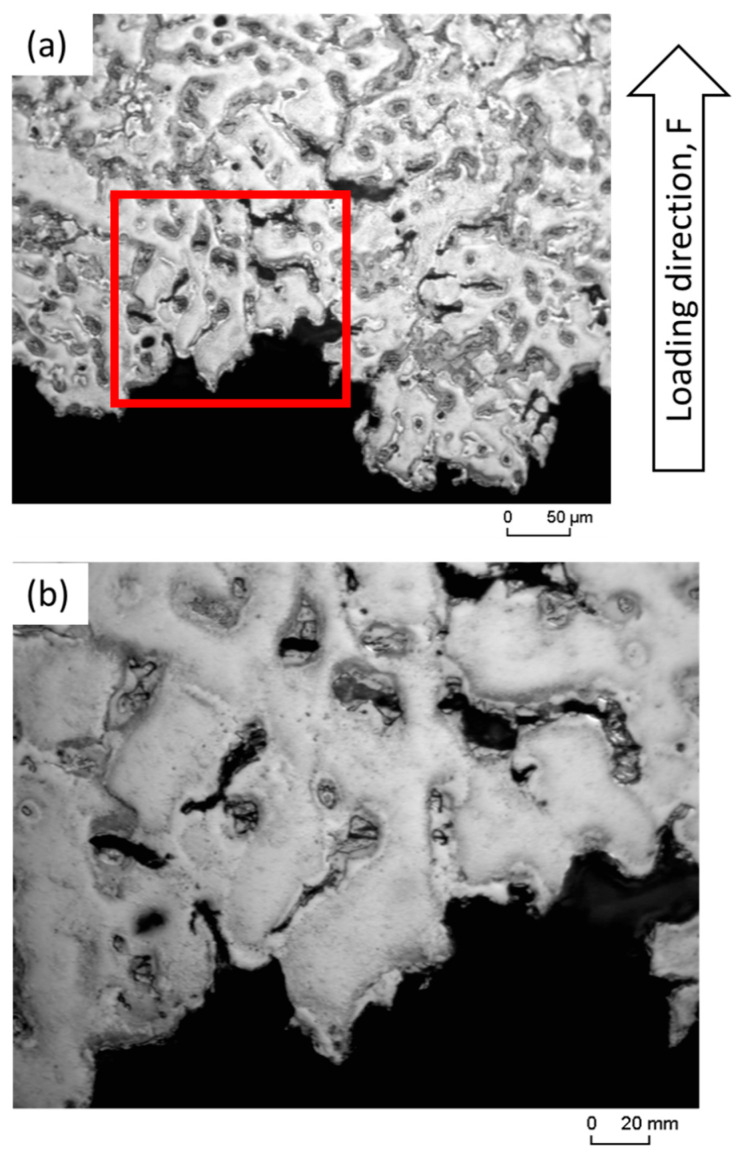
(**a**) Optical microstructure of a polished longitudinal section through tensile specimen of unreinforced AZ91 fractured at 20 °C and (**b**) higher magnified region of the image (**a**).

The images in Figure 10 demonstrate the optical microstructure of a polished longitudinal section through an unreinforced AZ91 tensile specimen that fractured at 20 °C. The existing fracture surface showed mild ductile to brittle behavior and was found to have cracks initiated by void coalescence. It can be seen that the fracture is dominated by the formation of voids that spread almost evenly throughout the deformed region (Figure 10a). Furthermore, voids form along grain boundaries and then play an important role in the fracture process. During the tensile test, the voids coalesced continuously, leading to microcracks initially; with continuous tensile load, the microcracks gradually transform into transverse cracks, which, at a later stage, reach the final fracture (Figure 10b). These microcracks tend to initiate at the brittle eutectic interfaces present along the grain boundaries, and it is these that cause the alloy to become brittle [51].

**Figure 11:**
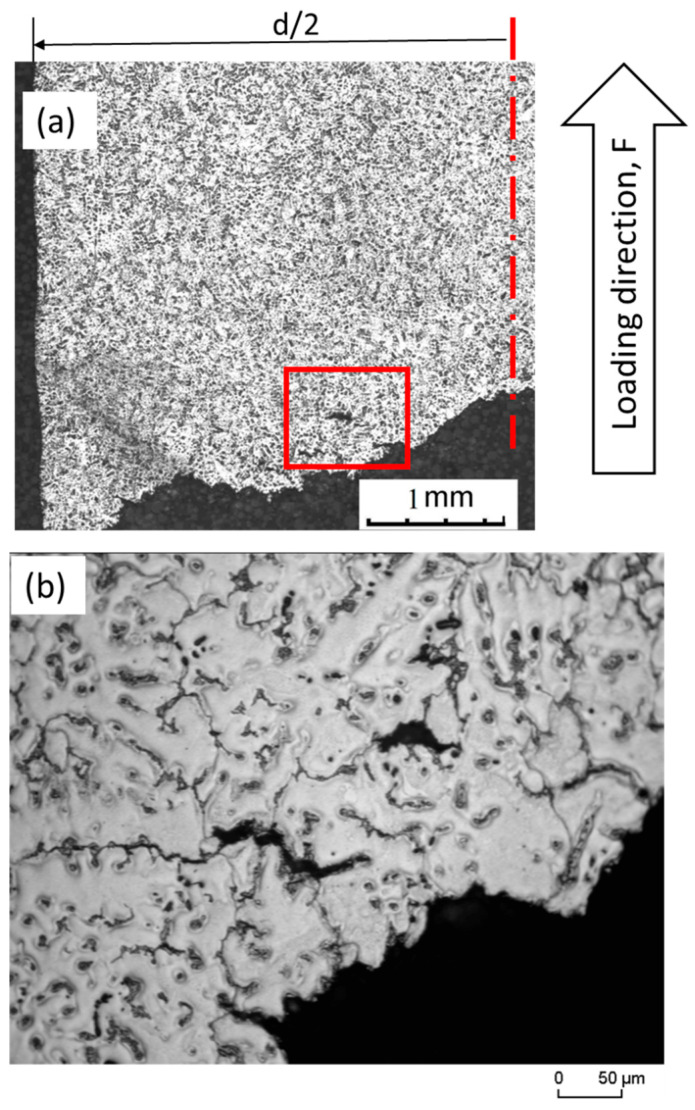
(**a**) Optical microstructure of a polished longitudinal section through tensile specimen of unreinforced AZ91 fractured at 150 °C and (**b**) higher magnification of the selected regions in image (**a**).

Figure 11 illustrates the optical microstructure of a polished longitudinal section through an unreinforced AZ91 tensile specimen that fractured at 150 °C. Due to the increased softening than that experienced at 20 °C, the fracture at 150 °C was more obvious than the fracture at 20 °C (Figure 10), where cracks and voids spread across the entire polished surface near the fracture (Figure 11a). It is clear that voids are formed along the grain boundaries, and when tensile testing is carried out, small voids condensate into large ones, which are the potential for the initiation of microcracks, which experience propagation, resulting in a river-like crack over time (Figure 11b). Briefly, relatively higher diffused voids and microcracks were detected in the fracture of the unreinforced AZ91 at a higher temperature.

**Figure 12:**
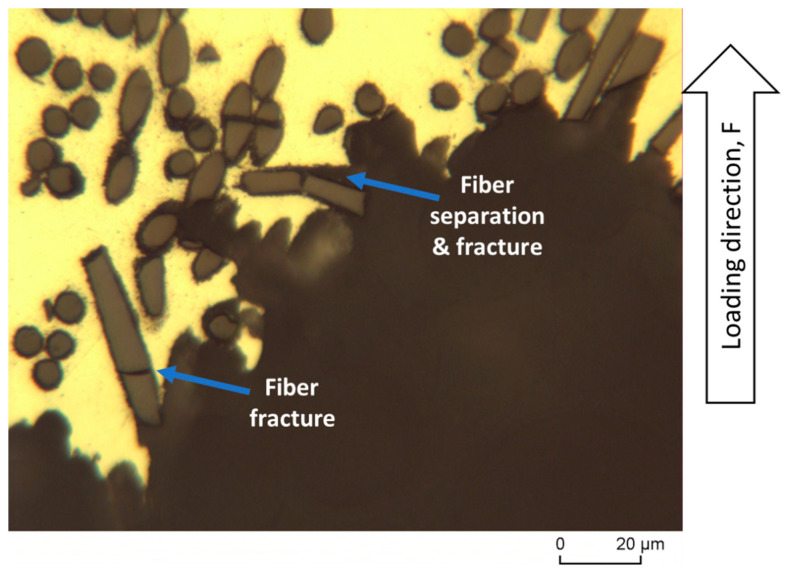
Optical microstructure of polished longitudinal section through tensile specimen of reinforced AZ91 fractured at 150 °C.

Figure 12 shows the optical microstructure of a polished longitudinal section through a reinforced AZ91 tensile specimen that fractured at 150 °C. By applying a tensile load, the fibers have the ability to withstand, due to the increase in load-carrying capacity by the reinforcement, which is then followed by accommodating themselves with some rotation and, concurrently, the matrix material shows some strain. Moreover, increasing the applied stress causes the short carbon fibers aligned parallel to the applied load to fracture. This is due to the low strain reaction of the short carbon fibers. Other fibers located perpendicular to the applied load suffer separation from the magnesium alloy matrix which eventually ends in fracture.

### 3.4. SEM Investigation Fracture Surface

**Figure 13:**
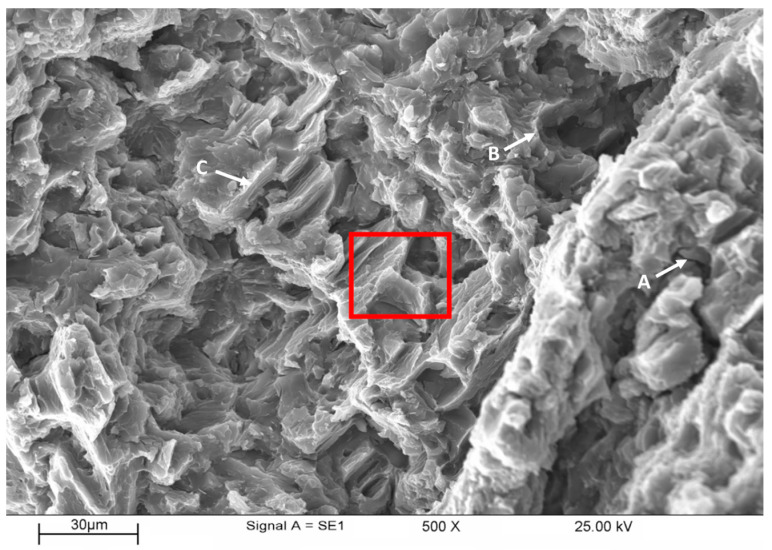
SEM of fracture surface of tensile specimen of the unreinforced AZ91 fractured at room temperature: (**A**) Cracks and cleavage planes; (**B**,**C**) Deformation walls which are potential sites for dimples. Red rectangle indicate the area magnified in Figure 14a.

Figure 13 shows the SEM of the fracture surface of a tensile specimen of unreinforced AZ91 fractured at room temperature. The SEM image reveals different forms of observable features and, for clarity, all of them are labeled in the micrograph. However, the fracture surface is mostly dominated by cleavages present in almost all parts of the micrograph surface. This is characteristic of a brittle intergranular cleavage fracture that occurred along grain boundaries [52]. The presence of dimples found in lower quantities than cleavages also further confirms that the fracture is mostly brittle.

**Figure 14:**
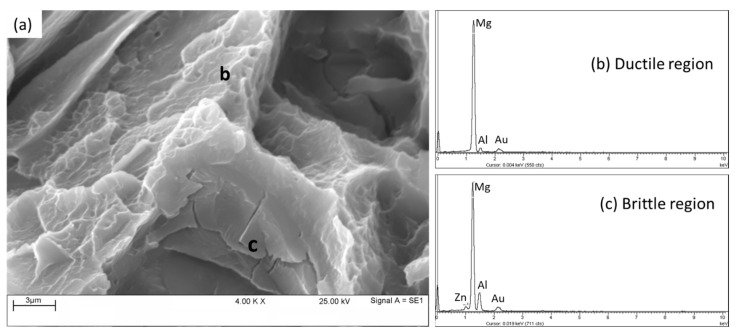
(**a**) Higher magnification of the above-highlighted region of fracture surface of tensile specimen of the unreinforced AZ91 fractured at room temperature; (**b**) EDX analysis of ductile region with dimples at position **b**, poor of Zn, and (**c**) EDX analysis of cleavage fracture with a crack in facets of the region having Zn at position **c**.

Figure 14 shows the fracture surface of a tensile specimen of unreinforced AZ91 at room temperature with a higher magnification of a selected region, highlighted by a square in Figure 13. It is clearly evident that the fracture surface is a combination of ductile and brittle regions which are labeled (b) and (c), respectively. From the visual observation of Figure 14a, the number of cleavages detected is greater than the dimples that can also be observed in some parts, meaning that the majority of the fault surface is still dominated by cleavages which make it brittle, while the dimples contribute to the formation of ductile regions. Further examination was conducted with EDS to determine the composition of each region that can be observed on the fracture surface. The results show that the ductile region consists of the Mg element, which has the maximum peak height, followed by the Al element at a lower concentration (Figure 14b). While the brittle region consists of Mg, which shows the strongest peak and is followed by Zn and Al at reasonable concentrations (Figure 14c). The presence of Zn can replace magnesium in the precipitated phase because both have the same valence electrons, leading to a relatively small electron concentration. In addition, Zn is close to Al in terms of its metallic radius, thus Zn may substitute Al. The presence of Zn can substitute the position of magnesium in the precipitate phase, which is an intermetallic phase because both have the same valence electrons, which causes a relatively small electron concentration. Additionally, Zn is close to Al in terms of metal radius. Consequently, Zn can replace Al. Based on the EDS results obtained, and results from previous studies [53], the possible intermetallic compounds formed refer to a Mg_17_Al_12_ phase for the ductile region and τ-Mg_32_(Al,Zn)_49_, ϕ- Mg_5_Al_2_Zn_2_, and MgZn_2_ phases for the brittle regions [53,54]. These Zn-rich compounds are considered as brittleness enhancing intermetallic compounds [54].

**Figure 15:**
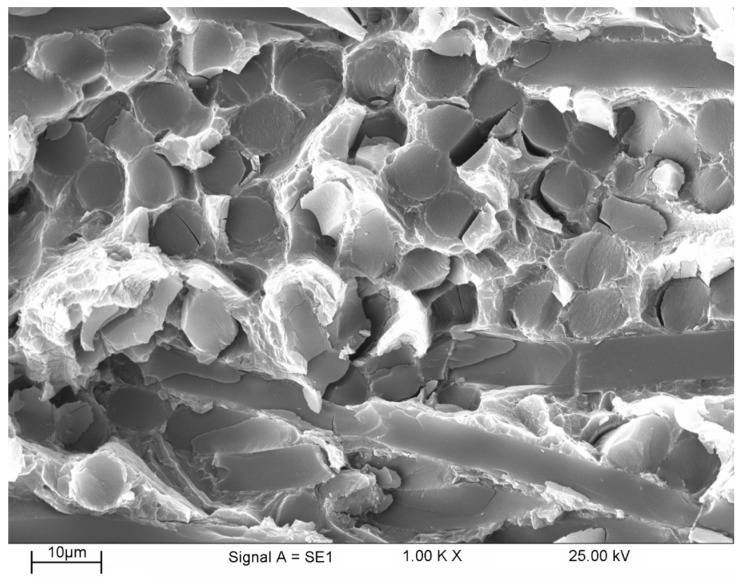
Fracture surface of reinforced AZ91-C tensile specimen fractured at room temperature.

Figure 15 shows the fracture surface of the tensile specimen of reinforced AZ91 broken at room temperature. From the fracture surface at room temperature, the homogeneous distribution of fibers in the matrix are obvious. It is also obviously found that most of the fiber fractures are parallel or oblique to the loading direction. In fact, fiber decohesion also took place in fibers that were parallel to the loading direction. The damage mechanisms detected started with several stages, namely, (i) fracture of the fibers, (ii) decohesion at the interface of the matrix with the fibers, and (iii) formation of cracks in the matrix. This condition continues until the crack reaches its critical point, which eventually leads to failure [53].

**Figure 16:**
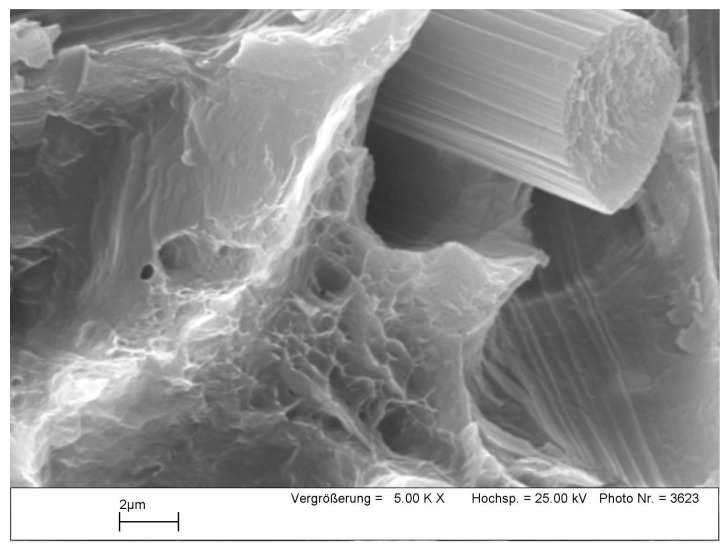
Higher magnification of a region from the fracture surface of tensile specimen of the reinforced AZ91 at room temperature.

Figure 16 illustrates a higher magnification of the fracture surface region of the reinforced AZ91-C tensile specimen at room temperature. There are a few important points to highlight, namely, the dimple region seen at the bottom, slightly to the left of the fiber position, and also the brittle region, which is also seen directly adjacent to the dimple region as indicated by the arrows. Of particular interest is the appearance of the fibers detaching from the matrix that binds them, resulting in the movement of the fibers out of their original place. The detachment of the carbon fibers was due to the poor interface between the carbon fibers and the matrix. A high-strength interface can be produced by chemical bonds formed through chemical reactions during composite fabrication [55]. However, excessive chemical reactions have the undesirable negative effect of damaging the integrity of the carbon fibers, which can reduce their ability to withstand the applied load [53].

## 4. Conclusions

Based on the results of the evaluation of tensile tests and description of the tensile flow curves at the temperature range from room temperature to 300 °C, as well as the microstructural and fracture investigation, the following conclusions can be drawn:

1. Magnesium alloy AZ91 is successfully reinforced by carbon short fiber with a volume fraction of V_f_ = 0.23 by squeeze casting.
2. The yield stress of AZ91 at 20 °C (109 MPa) is doubled (226 MPa) in the reinforced AZ91-C. The improvement of the yield stress due to reinforcing slightly decreases with increasing the test temperature.
3. The ultimate tensile strength of AZ91 at 20 °C (198 MPa) is increased to 262 MPa in the reinforced AZ91-C. The ultimate tensile strength of AZ91-C increases with increasing the test temperature.
4. Quasi-static tensile flow curves up to 300 °C are well described by the modified Mecking–Kocks method and the material parameters are determined as a function of the test temperature.
5. The fracture mode of the unreinforced AZ91 at room temperature is mixed with brittle cleavage fracture areas and a lower fraction of ductile deformation dimples.
6. The reinforced AZ91-C fracture is achieved by breaking the fibers parallel to the loading direction and detachment of the carbon fibers perpendicular to the line of loading from the matrix alloy (AZ91).

## 5. Future Work

As this reinforced AZ91-C material is planned to be a candidate for selectively reinforced truck pistons, and based on the above promising strengthening results, fatigue behavior of AZ91-C will be studied in future work.

## Figures and Tables

**Table 1:** General properties of the matrix alloy and the reinforcing fibers.

Components	Composition (wt. %)	Density (gm/cm^3^)	CTE (10^−6^ K^−1^)	Strength (MPa)	E-Modul (GPa)	Strain at Fracture ε (%)
Carbon Fibers (PAN) [31]	>95 % C L ≈ 92, d ≈ 7 µm	1.78	0.3 RT-100 °C	2000– 3500	225	1.2–1.5
AZ91	Mg—9 Al, 1 Zn	1.8	19–23 50–200 °C	240	45	9

**Table 2:** Composition of the matrix Mg-alloy AZ91.

Elements	Al	Zn	Si	Mn	Fe	Ni	Mg
wt. %	9.05	0.88	0.05	0.28	0.004	0.001	Rest

**Table 3:** Material law parameters as a function of the temperature depending on the values in Figure 9 and Equation (2).

Parameter	$k_{f}\;\left(T_{o\;}\right)(\mathrm{MPa})$	c (K/MPa)
ko	151	2.45
C1 = C3	22.4	−0.85
C2	118	0.878

## Data Availability

Data are available upon request through the corresponding author.
